# A Modified SDS-Based DNA Extraction Method for High Quality Environmental DNA from Seafloor Environments

**DOI:** 10.3389/fmicb.2016.00986

**Published:** 2016-06-23

**Authors:** Vengadesh Perumal Natarajan, Xinxu Zhang, Yuki Morono, Fumio Inagaki, Fengping Wang

**Affiliations:** ^1^State Key Laboratory of Microbial Metabolism, School of Life Sciences and Biotechnology, Shanghai Jiao Tong UniversityShanghai, China; ^2^State Key Laboratory of Ocean Engineering, Shanghai Jiao Tong UniversityShanghai, China; ^3^Guangdong Provincial Key Laboratory of Marine Biology, Marine Biology Institute, Shantou UniversityShantou, China; ^4^Geomicrobiology Group, Kochi Institute for Core Sample Research, Japan Agency for Marine-Earth Science and TechnologyKochi, Japan

**Keywords:** cell lysis efficiency, DNA extraction, DNA recovery, marine sediments, metagenomics, PCR

## Abstract

Recovering high quality genomic DNA from environmental samples is a crucial primary step to understand the genetic, metabolic, and evolutionary characteristics of microbial communities through molecular ecological approaches. However, it is often challenging because of the difficulty of effective cell lysis without fragmenting the genomic DNA. This work aims to improve the previous SDS-based DNA extraction methods for high-biomass seafloor samples, such as pelagic sediments and metal sulfide chimney, to obtain high quality and high molecular weight of the genomic DNA applicable for the subsequent molecular ecological analyses. In this regard, we standardized a modified SDS-based DNA extraction method (M-SDS), and its performance was then compared to those extracted by a recently developed hot-alkaline DNA extraction method (HA) and a commercial DNA extraction kit. Consequently, the M-SDS method resulted in higher DNA yield and cell lysis efficiency, lower DNA shearing, and higher diversity scores than other two methods, providing a comprehensive DNA assemblage of the microbial community on the seafloor depositional environment.

## Introduction

Microorganisms are ubiquitously distributed in various marine depositional environments, such as subseafloor continental shelves and hydrothermal deposits along the Mid-Ocean Ridges. The microbial community structure is always a fundamental question in understanding the ecological significance of their activities in biogeochemical elemental cycles. Because most members of the naturally occurring microbial community appeared to be resistant to cultivation in the laboratory (Rappe and Giovannoni, 2003), cultivation-independent molecular techniques, such as PCR-amplification of 16S rRNA genes followed by the high-throughput sequencing, provide us useful information of the microbial community. However, the molecular view of the microbial community could be significantly biased at the initial experimental step due to the incomplete recovery of total environmental DNA from the target microbial community (Cruaud et al., 2014; Luo et al., 2014; Morono et al., 2014).

Previous technological studies have developed or improved the DNA extraction method using various environmental samples, including aquifers, soils, and shallow seafloor deposits as well as deep subseafloor sediments (e.g., Martin-Laurent et al., 2001; Cruaud et al., 2014; Morono et al., 2014; Lever et al., 2015). The SDS-based environmental DNA extraction method standardized by Zhou et al. (1996) is one of the widely used DNA extraction methods in microbial ecology, which is applicable to various types of environmental samples. So far, many environmental DNA samples were prepared by this SDS method and used for the downstream molecular ecological analyses; e.g., microbial community structure (Knittel et al., 2005), functional gene (Wang et al., 2009) and metagenomics (Ettwig et al., 2010). Some studies further modified the SDS method with a bead-beating step (Biddle et al., 2006) or freeze-thawing step (Yeates et al., 1998; Lekang et al., 2015). The principal of this method is to use a high concentration of SDS for cell lysis, followed by adding chloroform-isoamyl alcohol to remove non-DNA biomolecules such as proteins and lipids, and then precipitating DNA with isopropanol. Others have developed a cryogenic mill based method which is appropriate for deep subseafloor sediments (Lipp et al., 2008); however, there were still challenging points in DNA recovery (Alain et al., 2011), shearing of DNA, and parallel processing of multiple samples.

In addition to the manual DNA extraction method with SDS, a number of commercial kits have been developed, which have also been widely used for the study of microbial communities. However, it has been often pointed out that cell lysis efficiency (Morono et al., 2014; Lever et al., 2015), DNA yield and quality (Miller et al., 1999; Kouduka et al., 2012), and biases of microbial community composition (Cruaud et al., 2014) largely depend on the sample type and method used for the experiment, resulting in varied data for the target microbial community. For example, because microbial communities in “deep” marine sediments have long been extremely difficult to prepare a comprehensive DNA, Morono et al. (2014) developed a hot-alkaline DNA extraction method that lysed over 98% of total cells in deep subseafloor sedimentary samples by using 1 M NaOH at 98°C for 20 min. The comparative study between HA and commercial kits showed that DNA extraction methods indeed could significantly impact on the indigenous microbial community composition, especially for archaeal communities of which cells might have rigid cell walls as compared to vegetative bacterial cells in the nutrient-rich shallow microbial habitats. Recently, Lever et al. (2015) reported a modular protocol for the DNA extraction and described that high SDS concentration and ethanol-NaCl precipitation have positive effect on the DNA yield. Taken together, the higher DNA yields and cell lysis efficiency for marine sedimentary communities were more often achieved with handcrafted methods than commercial kits, although commercial kits produced better comparable results especially by different individuals due to the method standardization. On the other hand, the high-molecular weight (large fragment) DNA is very valuable for the metagenomic sequencing and genomic binning, as it usually requires the construction of high quality DNA libraries prior to sequencing (Albertsen et al., 2013). Therefore, it is still necessary to improve and/or standardize the effective DNA extraction method to meet the analytical objectives on the target microbial community.

Quality and quantity of the recovered DNA may also vary depending on the applied DNA extraction method and the sample types. In general, examining cell lysis efficiency is a prior step to consider whether the applied DNA extraction method is suitable for the target sample. Various treatments, including physical, chemical and enzymatic cell lysis (such as bead-beating, SDS and/or proteinase K) are possible options for the protocol optimization (as summarized by Lever et al., 2015). It is a time-consuming step, but needs to be optimized for the limited amount of the target samples because of difficulties in collecting deep ocean samples. Purification and recovery of the DNA after cell lysis are other important steps that DNA loss and damage possibly occur (Roose-Amsaleg et al., 2001). Commonly used methods for DNA purification include the column purification (Howeler et al., 2003) and phenol:chloroform:isoamyl alcohol (24:25:1) extraction (Ogram et al., 1987). DNA loss during column purification has been often observed, which is most likely caused by competitive binding of humic substances to silica membranes (Lloyd et al., 2010). Charged minerals also affect the DNA extraction yield and stability by their binding with DNA and may prevent the complete dissolution of DNA in the extraction buffer (Barton et al., 2006; Vorhies and Gaines, 2009; Vishnivetskaya et al., 2014). The co-extracted humic compounds and heavy metal ions often hinder the downstream PCR-based molecular analyses (Tebbe and Vahjen, 1993; Wilson, 1997). Even worse, these co-extracted substances may damage DNA during the long-term storage and experimental time (Niemi et al., 2001; Zoetendal et al., 2001), which ultimately cause a significant bias of the DNA assemblage. Because samples from deep-sea and subseafloor environments are generally subjected to the limited amount, the optimized DNA extraction method with high DNA extraction yield and high cell lysis efficiency is always required in high demand.

Given those backgrounds described above, we tried to further improve the previous SDS-based DNA extraction method with the addition of a bead-beating step and a lysozyme incubation step for three representative seafloor samples, and then compared its performance with those processed by the HA method specifically optimized for deep subseafloor microbial (archaeal) communities (Morono et al., 2014) and a widely used commercial kit. We demonstrate here that the newly modified SDS-based DNA extraction method (M-SDS) recovered larger amount of the total genomic DNA with longer fragments, and could facilitate a better coverage of the indigenous microbial communities in those seafloor samples examined. Consequently, we confirmed that the M-SDS DNA extraction method provides high quality of the genomic DNA as a template for the subsequent metagenomic analysis.

## Materials and Methods

### Sample Description and Processing

Three samples were collected as representatives of deep-sea environments with relatively high biomass on the seafloor: (1) a microbial mat covered, organic rich oil-immersed hydrothermal sediment from the Guaymas Basin (herein GB), collected by *Alvin* with push corer during Cruise AT-15-25 dive 4460 in 2008; (2) a basaltic sulfide hydrothermal chimney from the East Pacific Rise (herein EPR), collected by *Jason* with robotic arm during Cruise AT-26 dive 10 in 2014; (3) a calcium carbonate and clay-rich seafloor sediment from the South China Sea (herein SCS), collected by gravity core during Cruise HYIV20130429 in 2013. Detailed information is provided in **Table 1**. Samples were stored at –80°C until use. For each whole-round sample, core section and chimney was subsampled with a sterile spatula in a laminar flow hood, and was further homogenized with agitation. In this study, in order to be in accordance with most of previous sample treatment procedures, extracellular DNA and intracellular DNA were not separated (Alawi et al., 2014) during DNA extraction.

**Table 1:** Summary of samples and the recovered DNA using three DNA extraction methods.

Sample location	Sample type	Temp (°C)	Depth (mbsf)	Water depth (m)	Cell abundance (cells/g sample)	DNA extraction method	DNA recovery (μg DNA/g sample)^1^	DNA recovery (μg DNA/g sample)^2^	Archaeal 16S rRNA gene copies/g sample	Bacterial 16S rRNA gene copies/g sample	Archaeal to Bacterial 16S rRNA gene ratio	Unlysed cell (cells/g sample)	Lysis efficiency (%)
Guaymas Basin (GB)	Oil-immersed hydrothermal sediment	31	0.06	∼2000	1.96 ± 0.22 × 10^10^	M-SDS	75.1 ± 6.95	12.95 ± 0.16	7.2 ± 0.2 × 10^9^	10.0 ± 0.1 × 10^8^	7.20	3.6 ± 0.6 × 10^8^	98.2


						HA	25.1 ± 3.4^3^	3.17 ± 0.14	1.5 ± 0.0 × 10^9^	3.5 ± 0.1 × 10^8^	4.26	2.7 ± 0.2 × 10^8^	98.6
						KIT	14.5 ± 3.5	0.25 ± 0.03	1.7 ± 0.2 × 10^6^	9.8 ± 0.7 × 10^5^	1.69	1.5 ± 0.3 × 10^9^	92.5
East Pacific Rise (EPR)	Basaltic sulfide, hydrothermal chimney	40	CS	2000	1.69 ± 0.25 × 10^9^	M-SDS	30.0 ± 1.41	1.21 ± 0.004	2.2 ± 0.3 × 10^7^	7.9 ± 0.1 × 10^7^	0.28	4.4 ± 0.02 × 10^7^	97.4


						HA	12.8 ± 0.3^3^	0.11 ± 0.03	3.4 ± 1.0 × 10^6^	4.7 ± 0.1 × 10^7^	0.07	2.9 ± 0.4 × 10^8^	82.9
						KIT	6.0 ± 0.77	0.06 ± 0.004	3.9 ± 0.2 × 10^4^	1.6 ± 0.1 × 10^6^	0.03	2.1 ± 0.5 × 10^7^	98.8
South China Sea (SCS)	Calcium carbonate, clay sediment	∼4	0.11	1600	1.17 ± 0.03 × 10^9^	M-SDS	43.2 ± 1.1	0.60 ± 0.01	3.1 ± 0.1 × 10^7^	1.6 ± 0.0 × 10^8^	0.19	1.1 ± 0.02 × 10^7^	99.0


						HA	23.3 ± 0.7^3^	0.12 ± 0.04	8.3 ± 0.3 × 10^6^	6.6 ± 0.2 × 10^7^	0.13	4.3 ± 0.8 × 10^7^	96.3
						KIT	8.2 ± 1.0	NM	8.3 ± 2.1 × 10^4^	3.1 ± 0.1 × 10^5^	0.26	5.9 ± 0.6 × 10^7^	94.9

*^1^DNA measured by spectrophotometry. ^2^DNA measured by Fluorometer method. ^3^DNA extracted by HA is regarded as single stranded DNA. CS, Chimney surface. NM, Not Measured duo to lack of DNA*.

### DNA Extraction Methods

**FIGURE 1:**
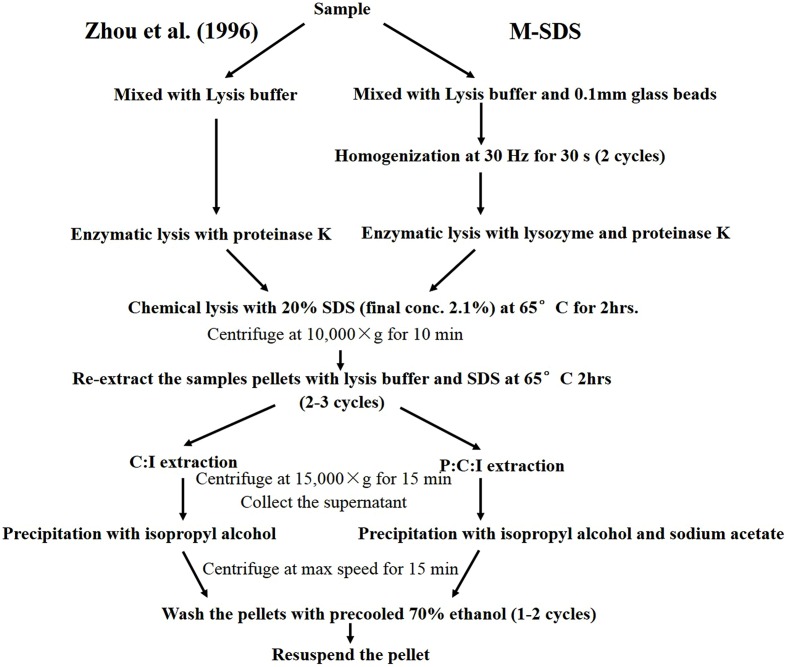
**Flow chart showing the modified SDS based-DNA extraction method (M-SDS)**. The right part is the M-SDS method used in this study. The left part is Zhou et al. (1996) method.

**FIGURE 2:**
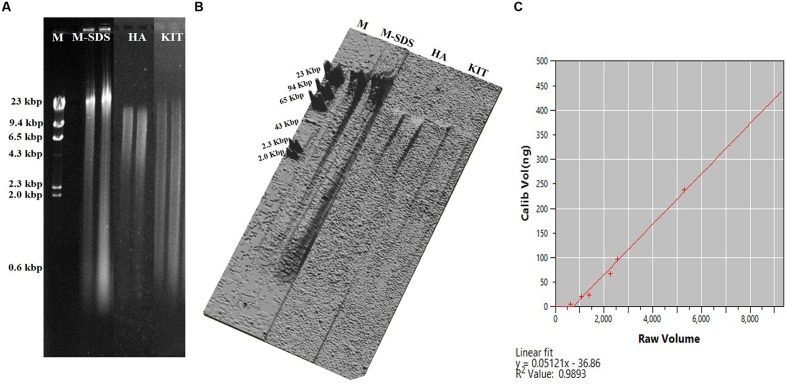
**(A)** Agarose gel electrophoresis of the recovered DNA from the Guaymas Basin sediment sample. Equal amount of sample (0.3 g) were extracted with three methods. The loading volume of the recovered DNA was 1 μL. M indicates Lambda DNA/HindIII Marker 2 (Thermo Fisher Scientific, USA). **(B)** Representation of DNA band intensity **(A)** as plotted in three dimensional image. **(C)** Standard curve for measuring the DNA concentration using CLIQS 1D Pro software as determined by the DNA band intensity on the agarose gel.

We tested three methods to extract DNA from seafloor samples: (1) a modified SDS-based method by Zhou et al. (1996; i.e., M-SDS; **Figure 1**); (2) a hot-alkaline method by Morono et al. (2014; i.e., HA); (3) a commercial kit (herein KIT). In general, 0.3 g of thoroughly mixed sample was used for each parallel extraction. For the DNA extraction with M-SDS, 0.3 g of a sediment or chimney sample was mixed with an equal weight of 0.1 mm-diameter glass beads and 670 μL of extraction buffer (100 mM Tris-HCl, 100 mM sodium EDTA, 100 mM sodium phosphate, 1.5 M NaCl and 10% cetyltrimethylammonium bromide, pH 8.0). The sample was mixed with the extraction buffer using low speed vortex for 5 min, and then homogenized with a tissue lyzer (Tissuelyser-48, Shanghai Jingxin, China) at 30 Hz for 30 s with a 120 s interval for two cycles. 50 μL lysozyme (20 mg/mL) and 10 μL proteinase K (20 mg/mL) was added and incubated for 30 min at a 37°C water bath. After incubation, 70 μL of 20% SDS was added and incubated at 65°C for 2 h with gentle mixing every 10 min. The supernatant was collected after 10,000 × *g* centrifuge for 10 min at room temperature, and transferred in a 2 mL microcentrifuge tube. The residual pellets were extracted once more by adding 500 μL extraction buffer and homogenized twice as described above, followed by adding 70 μL of 20% SDS and incubated at 65°C for 1 h, and then centrifuged. The supernatant from the two extraction steps were combined and mixed with an equal volume of phenol:chloroform:isoamyl alcohol (25:24:1 [vol/vol]). The aqueous phase was retained by centrifugation, and then the dissolved DNA was precipitated with ×0.6 volume of isopropyl alcohol and 0.3 M sodium acetate (pH 5.2) at 4°C overnight. The tube was centrifuged at 16,000 × *g* for 20 min at room temperature, and the supernatant was removed carefully to avoid the DNA loss. Finally, the pellet of DNA was washed with pre-cooled 70% ethanol and resuspended in 50 μL Milli-Q water.

The DNA extraction with HA, 0.3 g of sediment was mixed with pre warmed alkaline lysis solution (1 M NaOH, 5 mM EDTA pH 8.0 and 1% SDS) and incubate 70°C for 20 min. The incubated sediment samples were centrifuged at 1000 × *g* for 1 min at room temperature and supernatant was recovered and neutralized with 1 M HCl and 0.3 M Tris-HCl (pH 8.0). Then the sediment pellets were washed with pre-warmed double distilled water and recovered the solution by centrifugation then combined with previous supernatant. Combined supernatant solutions were treated with equal volume of phenol:chloroform:isoamyl alcohol and chloroform:isoamyl alcohol (25:1[vol/vol]) then the aqueous phase was collected by centrifugation. The Nucleic acid were precipitated by adding 0.1 volume of sodium acetate 3 M, ethachinamate and 2.5 times greater volume of ice-cold ethanol. The sample was then centrifuged at 15000 × *g* for 30 min at 4°C then washed the pellets with 70% ethanol and re-suspended with 1X TAE pH 8.0. The extracted DNA from the sediment was further purified using a spin column filled with polyvenylpolypyrrolidone (PVPP) to remove PCR inhibitors. PVPP preparation, purification and DNA was recovered according to Morono et al. (2014).

A commercial kit (PowerSoil DNA Isolation Kit, MO BIO Laboratories, Carlsbad, CA, USA) designed for soil DNA extraction was used in this study according to the manufacture’s protocol. Briefly, 0.3 g samples are added to a bead beating tube containing different size glass beads, lysis buffer for rapid and through homogenization with bead beating. Cell lysis occurs by mechanical and chemical method and the total genomic DNA were captured on a silica membrane in a spin column (column filtration). DNA were washed and eluted from the membrane. The DNA concentration was quantified by Nanodrop 2000 (UV/Vis. Spectrophotometer, Thermo Fisher Scientific, USA), and the fragment size was analyzed by electrophoresis on a 1% agarose gel. Since the DNA extracted using the HA method was regarded as single stranded DNA, the concentration was calculated by a spectrophotometer absorbance at 260 nm (Farrell, 2009; Morono et al., 2014). Double strand DNA (dsDNA) concentration was quantified by Qubit 3.0 fluorometer (Life Technologies, Invitrogen) using Qubit dsDNA HS Assay kit. And the DNA concentration was also compared by CLIQS 1D Pro software (TotalLab, UK) based on the intensity of the DNA bands on the agarose gel, using Lambda DNA/HindIII Marker 2 (Thermo Fisher Scientific, USA) as the standard curve. Different diameter of glass beads (0.1, 0.5 mm, mixed 0.1 and 0.5 mm at 1:1 ratio), the glass beads-to-sample ratio (0.5:1, 1:1, 1:2, 2:1), and the intensity of the tissue lyzer (20, 30, 40, 50, 70 Hz) were tested. A parallel blank extraction was performed during each batch of DNA extraction across three methods. The DNA extraction was considered free of contamination if no visual band was seen on the agarose gel for the PCR-amplified 16S rRNA genes.

### Cell Enumeration

Briefly, the seafloor samples before and after the DNA extraction were resuspended in 3% NaCl solution (2% paraformaldehyde were added as fixative). Microbial cell numbers and the cell lysis efficiency for each extraction method were calculated according to Morono et al. (2009, 2014).

### Quantification of Archaeal and Bacterial 16S rRNA Genes

The copy numbers of archaeal and bacterial 16S rRNA genes were quantified by quantitative PCR (qPCR) on Applied Biosystems 7500 Real Time system (Life Technologies, USA). The qPCR was performed with SYBR premix Ex Taq II (TaKaRa, Japan), 0.8 μM of each forward and reverse primers, and 1 μL of template DNA. The quantification, with a 25 μl reaction volume for each sample together with standard series and negative control, was run in triplicate. Archaeal 16S rRNA gene copies were quantified with primers Uni519F (5′-CAGCMGCCGCGGTAA-3′; Ovreas et al., 1997) and Arch908R (5′-CCCGCCAATTCCTTTAAGTT-3′; Jorgensen et al., 2012), with the thermal cycling program of 15 min at 95°C, and 40 cycles of 95°C for 30 s, 60°C for 30 s and 72°C for 45 s. *Escherichia coli* DH5α genomic DNA was used as the negative control. The standard curve was prepared using dilutions of PMD 18 T-Vector (TaKaRa, Japan) containing archaeal 16S rRNA gene fragments between 10^1^ and 10^7^. The amplification efficiency was 96%, and *R*^2^ of the standard curve was 0.99. Bacterial 16S rRNA gene copies were quantified with primers bac341F (5′-CCTACGGGWGGCWGCA-3′; Jorgensen et al., 2012) and 519r (5′-TTACCGCGGCKGCTG-3′; Ovreas et al., 1997), with the thermal cycling program of 15 min at 95°C, and 35 cycles of 95°C for 30 s and 72°C for 30 s. The standard curve consisted of a diluted PMD 18 T-Vector containing bacterial 16S rRNA gene fragments between 10^2^ and 10^7^. The genomic DNA of *Pyrococcus yayanosii* CH1 was used as the negative control. The amplification efficiency was 100%, and *R*^2^ of the standard curve was 0.99.

### PCR Amplification and Sequencing

The archaeal V4 region of the 16S rRNA gene was amplified with multi-tag primer sets U519F (5′-XXXXXXXXXXX YMGCC RCGGKAAHACC-3′) and Arch806R (5′-GGACTACNSGG GTMTCTAAT-3′; Song et al., 2013), where the X region represents various key tags for each sample. The PCR program was 3 min at 95°C, followed by 35 cycles of 94°C for 40 s, 56°C for 1 min and 72°C for 1 min. The final extension step was 72°C for 10 min. The bacterial V4 region the 16S rRNA gene was amplified by 520F (5′-XXXXXXXX AYTGGGYDTAAAGNG-3′) with eight-nucleotide key tags for each sample and 802R (5′-TACNVGGGTATCTAATCC-3′; Song et al., 2013). The 50 μL amplification mix contained 1 μL of each forward and reverse primer, 1 μL template DNA, 5 μL 10× Ex Taq buffer, 0.25 μL Ex Taq polymerase (TaKaRa, Japan), 4 μL of 2.5 mM dNTP mix and 5 μL of BSA (25 mg/mL). The PCR program was 3 min at 95°C, followed by 35 cycles of 94°C for 40 s, 56°C for 40 s and 72°C for 2 min. The final extension step was 72°C for 10 min. PCR products were purified by E.Z.N.A. Gel Extraction Kit (Omega Bio-Tek, USA) and sequenced on MiSeq platform (Illumina, USA) according to the manufacturer’s instructions.

### Microbial Community Composition and Statistical Analysis

A quality control step was applied to the raw sequence reads as described elsewhere (Zhang et al., 2016). Briefly, raw reads were removed if they contained a 50 bp continuous fragment with an average quality score less than 30 and/or any ambiguities. Filtered reads were merged together using FLASH (Magoč and Salzberg, 2011; Version 1.2.6). Merged sequences were removed if they contained more than six identical bases occurred continuously and/or any ambiguities, or the sequence length was <200 bp. Clean sequences were demultiplexed using the QIIME software pipeline (Caporaso et al., 2010; Version 1.9.0) with a mapping file containing the sample ID, barcode and primer sequence. Sequence reads were clustered into operational taxonomic units (OTUs) at 97% sequence similarity cutoff, and OTUs were assigned to the Greengenes database (DeSantis et al., 2006; Version gg_13_5) using the QIIME software pipeline (Caporaso et al., 2010; Version 1.9.0). Chimeras were detected with the UCHIME program (Edgar et al., 2011; Version 4.2) and removed from further analysis. Ternary plot, rarefaction analysis and diversity indices were performed using the PAST software package (Hammer et al., 2001). For the Venn diagram, sequences were rarefied to even depth (**Table 2**, the number of least sequences within each group was the SCS sample) by random sampling using QIIME. The Venn diagram was created using the Venn Diagram Plotter software^1^.

**Table 2:** Summary of archaeal and bacterial 16S rRNA gene sequences using three DNA extraction methods.

		Archaeal richness and diversity					Bacterial richness and diversity				
Sample	DNA extraction method	Number of sequences	Number of OTUs (97%)	Richness (ACE)	Shannon’s index (Chao and Shen)	Simpson index (MLE)	Number of sequences	Number of OTUs (97%)	Richness (ACE)	Shannon’s index (Chao and Shen)	Simpson index (MLE)
Guaymas Basin (GB)	M-SDS	42,872	1,563	0.71	1.69	0.09	23,505	398	0.69	2	0.04


	HA	39,798	682	0.63	1.35	0.07	23,471	398	0.68	1.75	0.04
	KIT	9,587	572	0.71	1.52	0.09	19,730	338	0.53	1.44	0.03
East Pacific Rise (EPR)	M-SDS	46,773	639	0.83	2.26	0.15	29,766	1,171	0.97	4.18	0.22


	HA	44,958	439	0.81	2.23	0.16	25,722	1,030	0.94	3.6	0.14
	KIT	37,700	513	0.76	2.07	0.16	22,126	894	0.94	3.54	0.13
South China Sea (SCS)	M-SDS	56,410	953	0.81	2.16	0.19	25,116	2,072	0.91	3.47	0.10


	HA	33,668	680	0.79	2.02	0.15	18,569	1,711	0.89	2.96	0.13
	KIT	30,874	676	0.72	1.95	0.12	14,105	1,684	0.84	2.53	0.11

*ACE indicates abundance-based coverage. MLE indicates Maximum likelihood estimator*.

### Metagenome Sequencing and Community Composition Analysis

Approximately 5 μg DNA extracted from the GB sample by M-SDS was used for metagenome sequencing by BGI-Shenzhen. The DNA was fragmented and subjected to gel-electrophotometry. An end repair mix was added to the fragmented DNA, and the mixture incubated at 20°C for 30 min and then purified with QIAquick PCR Purification Kit (Qiagen, Germany). After that, A-tailing mix was added and the mixture was incubated at 37°C for 30 min. Together with adapter and ligation mix, the purified 3′ ends adenylate DNA mixture was incubated for the ligation reaction at 20°C for 15 min. The adapter-ligated DNA was recovered from 2% agarose gel. The selected gel was purified with QIAquick Gel Extraction kit (Qiagen, Germany), and a few rounds of PCR amplification were conducted to enrich the adapter-ligated DNA fragments. Another round of 2% agarose gel was conducted to recover the target fragments. The libraries with a 350 bp-insert size were amplified to generate a cluster on the flowcell (TruSeq PE Cluster Kit V3KcBot–BotCIllumina). Pair-end sequencing of the amplified flowcell was performed using HiSeq 2000 System (TruSeq SBS KIT-HS V3, Illumina, at BGI-Shenzhen). The raw metagenomic reads obtained by Illumina pair-end sequencing were dereplicated (100% identity over 100% lengths) and trimmed using sickle^2^. 16S rRNA sequences were extracted using Sortmerna (Kopylova et al., 2012) and mapped to OTUs based on clustering of reference 16S sequences (Quast et al., 2013) at 97% sequence similarity cutoff.

### Accession Number

All sequence data have been deposited in the National Center for Biotechnology Information (NCBI) Sequence Read Archive under the accession number SRP072161.

## Results and Discussion

### Microbial Abundance

Microbial concentration in the GB sediment sample was 1.96 × 10^10^ cells/g as determined by fluorescence microscopy (**Table 1**). This sample was rich in hydrocarbons (oil-immersed), which were derived from terrestrial organic matter and hydrothermal activity. The hydrothermal chimney sample from EPR was rich in silicate, sulfide and iron minerals, and its cell concentration was 1.69 × 10^9^ cells/g. The SCS pelagic sediment sample contained ∼1.4 wt% of organic matter (Wang et al., 2010), and its cell concentration was 1.17 × 10^9^ cells/g. The qPCR quantification of the bacterial and archaeal 16S rRNA gene copy numbers indicated that the GB sample was dominated by archaea while bacteria dominated EPR and SCS samples (**Table 1**).

### DNA Recovery

The M-SDS method recovered the maximum DNA amount among the three different seafloor samples, which was up to ∼4 times higher than the other two methods (**Table 1**). This result was further confirmed by fluorometric method and quantification of the DNA concentration based on the band intensity on the agarose gel (**Figure 2**). Compared with other two methods, longer fragments of the total DNA were recovered by M-SDS, showing a clear main band on the agarose gel (>23 kb) despite some DNA fragmentations occurred. We should emphasize that the focus of this study is DNA extraction from seafloor samples where relatively high biomass are present. Although, we also tested DNA extraction from low-biomass subsurface sediment samples (∼10^3-4^ cells/g sediment), the DNA quantity obtained by all methods was below quantification limit, therefore no conclusion could be made and not included in the study.

The highest yield of DNA was achieved by M-SDS possibly due to the combination of multiple cell lysis treatments, including physical (bead-beating), chemical (SDS surfactant), and enzymatic (proteinase K and lysozyme) steps. During the modification of Zhou’s SDS-based DNA extraction method, the addition of a bead-beating step always got higher DNA yields compared to cell lysis treatments tested in this study (i.e., sonication and freeze-thaw). Particularly, the bead-beating step was carefully modified with different conditions, including the intensity of the tissue lyzer, the size of the glass beads, and the ratio of glass beads to sample, resulting in the best optimized condition (higher DNA yield and longer fragment) of 30 Hz intensity, 0.1 mm diameter glass bead, and 1:1 of the bead to sample ratio (**Figure 1**). Moreover, a twice of ethanol-wash step was used in the final purification instead of using the column filtration (as used by HA and KIT), which may further reduce the chance of DNA breakdown and loss. Substantial DNA loss may occur during silica column purification due to the competitive binding of co-extracts (such as humic acid) to silica membranes, which was not used for all three methods tested in this study (Howeler et al., 2003; Lloyd et al., 2010; Lever et al., 2015). DNA extracts without the final column purification step by HA and KIT methods could not be amplified by PCR (data not shown). The ethanol-washed DNA could be further used for PCR-amplification and metagenomic sequencing without any additional purification steps.

The environmental DNA extracted by the M-SDS method was characterized by higher yields and longer fragments (**Figure 2**; **Table 1**). It was proved suitable for the metagenomic study since a high quality metagenomic sequence assemblage was created from the GB sample (see below for more details). In contrast, highly fragmented DNA were obtained by HA and KIT methods (**Figure 2**), indicating that DNA was physically and/or chemically broken during the extraction steps. For the HA method, DNA was denatured into single strand and might have lost its structural stability due to the high alkaline condition (Ageno et al., 1969; Kouduka et al., 2012). In addition, the high temperature treatment (70°C for 20 min) might damage DNA, making the length shorter than 23 kb as reported previously (Morono et al., 2014). The KIT method also yielded less concentrated DNA with relatively short fragments.

The copy numbers of archaeal and bacterial 16S rRNA genes recovered from GB, EPR, and SCS samples by three DNA extraction methods were determined by qPCR. M-SDS recovered the highest 16S rRNA gene copies both for archaea and bacteria, which was consistent with the highest DNA yield (**Table 1**). In the GB sample, for example, the M-SDS method obtained 7.19 × 10^9^ archaeal 16S rRNA gene copies/g and 9.98 × 10^8^ copies/g for bacteria, which were 3–5 folds higher than those in DNA extracted by HA, and >10^3^ folds higher than those using the commercial kit. Surprisingly, the number of archaeal and bacterial 16S rRNA gene copies obtained by KIT was approximately 2–3 orders of magnitude lower than those by M-SDS and HA. This was possibly attributed to its substantial loss of DNA during extraction steps, together with the fragmented DNA, which finally reduced the number of 16S rRNA gene templates for the qPCR analysis.

In addition, a higher archaeal to bacterial 16S rRNA gene copy ratio was observed by the M-SDS method from GB (7.2) and EPR (0.28) samples. Since archaeal cell membrane is generally more rigid than bacteria (Valentine, 2007), they are overall highly resistant to cell lysis for the DNA extraction. Nevertheless, M-SDS had a better performance of recovering archaeal DNA, which would provide less-biased archaeal community data. It has noted that harsher extraction methods sheared the bacterial DNA (Zhou et al., 1996; de Lipthay et al., 2004), which may cause the higher ratios of archaea vs. bacteria in the samples. However, this would not be the case here, as the M-SDS method recovered long, less-sheered DNA. This is important for microbial communities inhabiting various seafloor environments, where archaea tend to play a significant role in energy metabolisms and elemental cycles (Lloyd et al., 2013; Meng et al., 2014; Vigneron et al., 2014).

### Cell Lysis Efficiency

Cell lysis efficiency was determined by comparing cell concentrations in the sample before and after DNA extraction. In general, between 82.9 and 99.0% of lysis efficiencies were obtained by using three methods examined, although different samples resulted in varied lysis efficiencies (**Table 1**). In the GB sample, HA and M-SDS lysed 98.6 and 98.2% of the total microbial cells, respectively. These results were slightly higher than that with the KIT (92.5%). The highest cell lysis efficiency was observed in the HA method (98.6%), indicating the advantage of hot-alkaline incubation for archaea-dominated samples or deep subseafloor sediments as reported previously (Morono et al., 2014). For the EPR chimney sample, however, the cell lysis efficiency by the HA method was 82.9%, although KIT and M-SDS methods gave the better performance of cell lysis efficiency, which were 98.8 and 97.4%, respectively. This may be likely due to the dissolution of high metal contents in the chimney sample (James et al., 2014), thus changing the extraction buffer pH or inactivating the lysing enzymes. For example, (1) high concentrations of iron and manganese ions may form hydroxide precipitates and lower the buffer pH; (2) heavy metals may cause the inactivation of proteinase K and lysozyme. For the SCS sediment sample, the M-SDS method gave the best performance of cell lysis efficiency (99.0%), followed by HA (96.3%) and KIT (94.9%). The SCS sample was characterized by abundant clay particles (Liu et al., 2010), which may adsorb a significant amount of the extracted DNA (Barton et al., 2006). The high cell lysis efficiency obtained by M-SDS was possibly due to the extraction buffer contained more anionic and cationic surfactants, which might help to isolate the adsorbed DNA from the charged clay minerals (Dias et al., 2004, 2008; Rosa et al., 2005).

### Comparison of Microbial Communities

The highest number of archaeal and bacterial 16S rRNA gene sequences were obtained by M-SDS, followed by HA and KIT, although the equal amount of PCR-amplified DNA were sequenced in the same run (**Table 2**). The reason is not clear yet, but it was suggested that higher quality of DNA helps to increase the target amplification percentage and reduces the chances of unspecific amplification and chimera formation, thus producing more qualified amplicons for sequencing (Martin-Laurent et al., 2001; Scupham et al., 2007; Sergeant et al., 2012).

**FIGURE 3:**
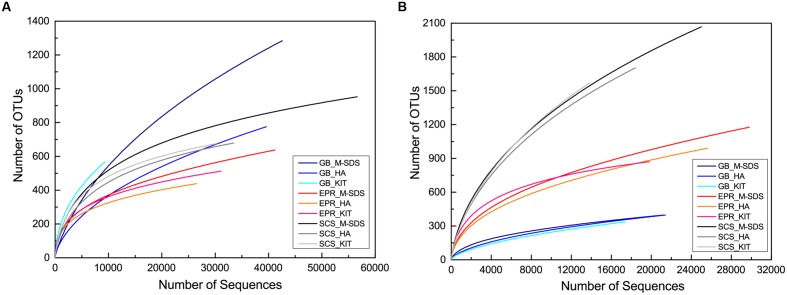
**Rarefaction curves for **(A)** archaeal and **(B)** bacterial 16S rRNA gene sequences obtained from three seafloor samples by three different DNA extraction methods**. Sequences were clustered into OTUs at 97% similarity cutoff. X and Y axis are not presented on the same scale.

**FIGURE 4:**
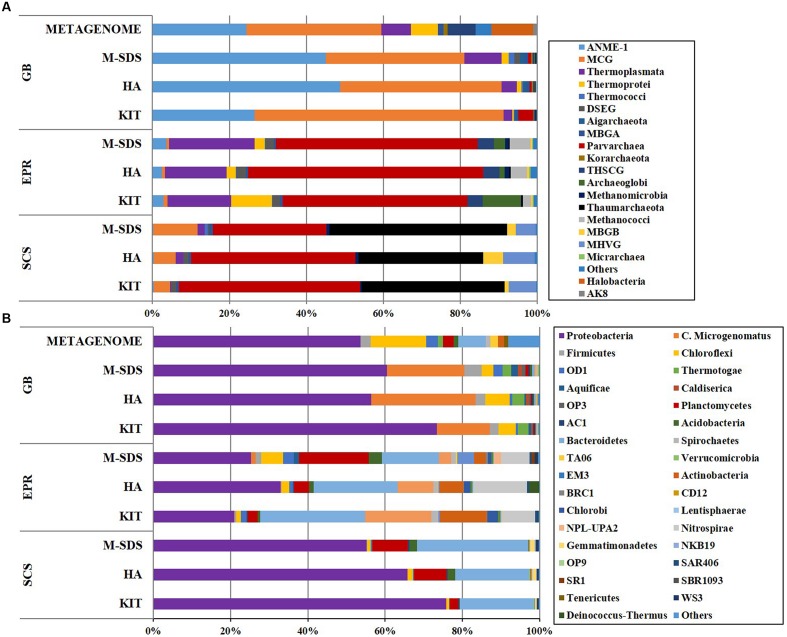
**(A)** Archaeal (class level) and **(B)** bacterial (phylum level) taxonomic diversity of microbial communities in three seafloor samples obtained by three different DNA extraction methods. Color bars indicate the percentage of the designated group within each sample. Only >0.1% abundance are listed. The remaining sequences are grouped to “Others.”

The rarefaction curve of bacterial communities indicated that most bacterial taxa have been covered at this sequencing depth, although the SCS sediment sample did not plateau (**Figure 3**). However, the archaeal rarefaction curve gave varied results. As expected, the microbial community structure determined by three DNA extraction methods shared much similarity in each sample. In the GB sediment sample, *Bathyarchaeota* (formerly referred as Miscellaneous Crenarchaeota Group [MCG]) and ANMEs were predominant in the archaeal community, followed by *Thermoplasmata* and *Parvarchaea*. Compared to other two methods, the KIT method recovered higher ratios of *Bathyarchaeota* (64%) and lower ratios of ANMEs (26%; **Figure 4**). For the bacterial community, *Proteobacteria*-related sequences were also enriched from the KIT method. The M-SDS and HA methods resulted in similar microbial communities, showing predominance of ANME-1, *Thermoplasmata* for Archaea, and *Candidatus microgenomatus* and *Firmicutes* for Bacteria. It is reported that ANME groups often occurred as the aggregate which are covered by thick extracellular polymeric substances as well as carbonates and encrusted minerals (Boetius et al., 2000; Knittel and Boetius, 2009; Chen et al., 2014). The combination of bead-beating, SDS surfactant, and enzymatic lysis steps in the M-SDS protocol helps to (1) remove cells from the aggregated consortium with minerals (Miller et al., 1999; Niemi et al., 2001; Luna et al., 2006; Morono et al., 2014), and (2) break the rigid cell wall of gram-positive bacteria, likely resulting in higher ratios of ANME- and *Firmicutes*-related sequences. The similar trend was also seen from EPR and SCS samples. These observations suggest that M-SDS and HA methods may have a superior performance of isolating some archaeal or aggregated microbial communities. For the EPR sample, *Parvarchaeota* (48.0–61.1%) and *Thermoplasmata* (16.5–22.2%) dominated the archaeal community, whereas *Proteobacteria* (20.9–32.9%) and *Bacteroidetes* (14.8–27.1%) dominated the bacterial community. For the SCS sample, major groups are *Parvarchaeota* (29.5–47.2%), *Thaumarchaeota* (32.5–46.2%), followed by minor groups such as *Bathyarchaeota* (4.2–11.5%) and MHVG (5.1–8.3%), whereas bacterial communities were dominated by *Proteobacteria* (55.0–80.18%) and *Bacteroidetes* (19.1–28.6%).

The highest archaeal and bacterial diversity indices and the total number of OTUs were observed by the M-SDS method, followed by HA and KIT (**Table 2**). The ternary plot for archaeal and bacterial communities showed that most of the major groups (>10%) in each sample were homogeneously distributed by using three DNA extraction methods (Supplementary Figure S1). This result indicates that the three DNA extraction methods are appropriate for identifying major microbial groups. Notably, the M-SDS method showed an outstanding performance when microbial groups with relative abundance less than 1% in the total microbial community were of research interest (**Figure 4**). For example, the ternary plot of archaeal 16S rRNA genes from the GB sample showed a better recovery of members within the Deep Sea Euryarchaeotal Group (DSEG), *Thermococci*, *Thermoplasmata*, *Thermoprotei*, and *Archaeoglobi* (Supplementary Figure S1A); for bacterial 16S rRNA genes, the M-SDS method showed best performance of recovering bacterial minor groups from the EPR sample (SR1, NPL-UPA2, WS3, *Candidatus microgenomatus*, LD1, TA06, *Planctomycetes*, etc.; Supplementary Figure S1B).

**FIGURE 5:**
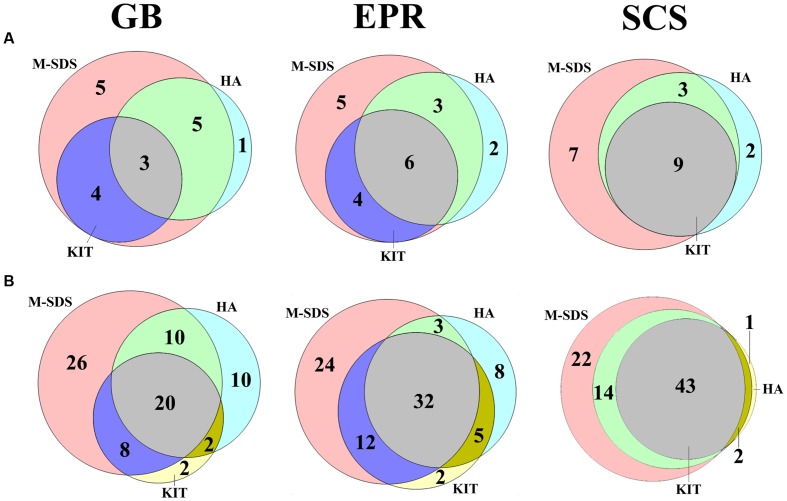
**Venn diagram of **(A)** archaeal (order level) and **(B)** bacterial (class level) rare biosphere**. The rare biosphere indicates the abundance of the designated group is <0.1%. Values in the circles indicate the number of detected groups.

We analyzed archaeal and bacterial taxa with the relative abundance less than 0.1%, which assemblage is called “rare biosphere” (Sogin et al., 2006; Lynch and Neufeld, 2015). As shown in **Figure 5A**, M-SDS had an overall higher coverage over KIT at archaeal order level, and only 1–2 archaeal taxa detected by HA did not overlap with M-SDS. HA and KIT roughly overlapped half of their total rare components in GB and EPR samples, although the groups obtained by KIT was overlain by HA in the SCS sample. The similar trend was observed for bacterial communities at class level (**Figure 5B**). The M-SDS method retrieved a higher overall coverage of rare bacterial community members than HA and KIT in three samples, resulting in the detection of some unique bacterial taxa (i.e., 26 in GB, 24 in EPR, and 22 in SCS at class level). In summary, the M-SDS method detected the most diverse archaeal and bacterial components, and thus was found to be suitable for those three samples to extract DNA for studies targeting on the rare biosphere. As discussed previously, we attributed this to the long and intact fragments and high recovery of DNA by M-SDS.

A metagenome from the GB sediment sample was constructed using DNA extracted by the M-SDS method using Hiseq 2000 platform for sequencing (conducted at the BGI-Shenzhen, China). The generated GB metagenome contained ∼30 Gbp raw data, from which metabolic potentials of the microbial community will be investigated in the future. From the metagenomic sequence pool, a total of 126primer,857 16S ribosomal RNA gene tags (Logares et al., 2014) were extracted and mapped to OTUs based on clustering of reference in Silva database, of which 70,704 were assigned to bacterial class and 56,153 to archaeal class. The archaeal community was dominated by *Bathyarchaeota* (35%), ANME-1 (24%), *Halobacteria* (11%) and *Thermoplasmata* (8%), while *Proteobacteria* (50%), *Chloroflexi* (13%), *Bacteroidetes* (7%) dominated the bacterial community (**Figure 4**). The similar taxonomic compositions of archaeal and bacterial communities were observed by PCR-amplified 16S rRNA genes from the GB sample. However, *Candidatus microgenomatus* was found to constitute ∼20% of the total bacterial community by PCR, while depleted in the metagenome (**Figure 4**). This may be possibly due to the bias caused by PCR primers, metagenomic sequences (Teske and Sorensen, 2007; Pinto and Raskin, 2012; Cruaud et al., 2014). We had a preliminary check of the PCR primers used in this study toward the *Candidatus microgenomatus* 16S rRNA gene sequences in the databank, no clear bias toward *Candidatus microgenomatus* was noticed. Metagenomic data recovered higher microbial diversity than PCR at different taxonomic levels (phylum, class, order, and family) in most abundant taxa (Supplementary Figure S2A). The recovery of rare components in the metagenomic pool was also up to 50 times higher for archaea and 30 times higher for bacteria at each taxonomic level (Supplementary Figure S2B). This is likely because metagenomic sequences can cover any region of the 16S rRNA genes but the PCR-based method only cover a specific region (Nyyssönen et al., 2013).

## Conclusion

This study demonstrated that the improved SDS-based DNA extraction method (i.e., M-SDS) can recover high yield and low shearing genomic DNA from three representative seafloor samples. Together with the high cell lysis efficiency, the M-SDS method facilitates a better coverage of the total microbial community, including minor components of the rare biosphere. However, it should be noted that the kit method is faster and easier in manipulation, it’s still valuable and recommended when the main components of the community are targeted and/or large amount of samples are under screening. Because high quality genomic DNA were obtained with high cell lysis efficiencies, DNA extracted by M-SDS is applicable for various downstream molecular analyses, including metagenomics. It should be emphasized that the M-SDS method does not always guarantee the best performance to remarkably diverse microbial habitats on our planet; e.g., DNA extraction from some forms of microbial cells under extremely energy-limiting conditions (e.g., the deep biosphere) or in specific geologic samples requires the carefully optimized physical and/or chemical treatments like the HA and other methods for each target, which is still a challenging technological development. Meanwhile, in order to guarantee DNA extraction efficiency and minimize bias, multiple extraction protocols should be applied in parallel and the recovered DNA should be pooled for downstream sequencing applications

## Author Contributions

FW and FI: designed the experiments and analyzed the data; VN: performed the experiments for nucleic acid extractions, quantitative PCR and 16S rRNA gene sequencing. XZ: analyzed the data and wrote the paper. YM: performed the cell enumeration. XZ, VN, and FW: wrote the manuscript in consultation with all other authors.

## Conflict of Interest Statement

The authors declare that the research was conducted in the absence of any commercial or financial relationships that could be construed as a potential conflict of interest.

## Footnotes

1 http://omics.pnl.gov/software/venn-diagram-plotter

2 https://github.com/najoshi/sickle

## References

[B1] Ageno M. Dore E. Frontali C. (1969). The alkaline denaturation of DNA. *Biophys. J.* 9 1281–1311. 10.1016/s0006-3495(69)86452-0 4982056 PMC1367631

[B2] Alain K. Callac N. Ciobanu M. C. Reynaud Y. Duthoit F. Jebbar M. (2011). DNA extractions from deep subseafloor sediments: novel cryogenic-mill-based procedure and comparison to existing protocols. *J. Microbiol. Methods* 87 355–362. 10.1016/j.mimet.2011.09.015 22005039

[B3] Alawi M. Schneider B. Kallmeyer J. (2014). A procedure for separate recovery of extra- and intracellular DNA from a single marine sediment sample. *J. Microbiol. Methods* 104 36–42. 10.1016/j.mimet.2014.06.009 24955890

[B4] Albertsen M. Hugenholtz P. Skarshewski A. Nielsen K. L. Tyson G. W. Nielsen P. H. (2013). Genome sequences of rare, uncultured bacteria obtained by differential coverage binning of multiple metagenomes. *Nat. Biotechnol.* 31 533–538. 10.1038/nbt.2579 23707974

[B5] Barton H. A. Taylor N. M. Lubbers B. R. Pemberton A. C. (2006). DNA extraction from low-biomass carbonate rock: an improved method with reduced contamination and the low-biomass contaminant database. *J. Microbiol. Methods* 66 21–31. 10.1016/j.mimet.2005.10.005 16305811

[B6] Biddle J. F. Lipp J. S. Lever M. A. Lloyd K. G. Sorensen K. B. Anderson R. (2006). Heterotrophic Archaea dominate sedimentary subsurface ecosystems off Peru. *Proc. Natl. Acad. Sci. U.S.A.* 103 3846–3851. 10.1073/pnas.0600035103 16505362 PMC1533785

[B7] Boetius A. Ravenschlag K. Schubert C. J. Rickert D. Widdel F. Gieseke A. (2000). A marine microbial consortium apparently mediating anaerobic oxidation of methane. *Nature* 407 623–626. 10.1038/35036572 11034209

[B8] Caporaso J. G. Kuczynski J. Stombaugh J. Bittinger K. Bushman F. D. Costello E. K. (2010). QIIME allows analysis of high-throughput community sequencing data. *Nat. Methods* 7 335–336 10.1038/nmeth.f.303 20383131 PMC3156573

[B9] Chen Y. Li Y. L. Zhou G. T. Li H. Lin Y. T. Xiao X. (2014). Biomineralization mediated by anaerobic methane-consuming cell consortia. *Sci. Rep.* 4:5696 10.1038/srep05696 PMC4100016 25027246

[B10] Cruaud P. Vigneron A. Lucchetti-Miganeh C. Ciron P. E. Godfroy A. Cambon-Bonavita M. A. (2014). Influence of DNA extraction method, 16S rRNA targeted hypervariable regions, and sample origin on microbial diversity detected by 454 pyrosequencing in marine chemosynthetic ecosystems. *Appl. Environ. Microbiol.* 80 4626–4639. 10.1128/AEM.00592-14 24837380 PMC4148798

[B11] de Lipthay J. R. Enzinger C. Johnsen K. Aamand J. Sørensen S. J. (2004). Impact of DNA extraction method on bacterial community composition measured by denaturing gradient gel electrophoresis. *Soil Biol. Biochem.* 36 1607–1614. 10.1016/j.soilbio.2004.03.011

[B12] DeSantis T. Z. Hugenholtz P. Larsen N. Rojas M. Brodie E. L. Keller K. (2006). Greengenes, a chimera-checked 16S rRNA gene database and workbench compatible with ARB. *Appl. Environ. Microbiol.* 72 5069–5072. 10.1128/AEM.03006-05 16820507 PMC1489311

[B13] Dias R. Rosa M. Pais A. C. Miguel M. Lindman B. (2004). DNA-Surfactant interactions, compaction, condensation, decompaction and phase separation. *J. Chin. Chem. Soc.* 51 447–469. 10.1002/jccs.200400069

[B14] Dias R. S. Magno L. M. Valente A. J. Das D. Das P. K. Maiti S. (2008). Interaction between DNA and cationic surfactants: effect of DNA conformation and surfactant headgroup. *J. Phys. Chem. B* 112 14446–14452. 10.1021/jp8027935 18774843

[B15] Edgar R. C. Haas B. J. Clemente J. C. Quince C. Knight R. (2011). UCHIME improves sensitivity and speed of chimera detection. *Bioinformatics* 27 2194–2200. 10.1093/bioinformatics/btr381 21700674 PMC3150044

[B16] Ettwig K. F. Butler M. K. Le Paslier D. Pelletier E. Mangenot S. Kuypers M. M. M. (2010). Nitrite-driven anaerobic methane oxidation by oxygenic bacteria. *Nature* 464 543–548. 10.1038/nature08883 20336137

[B17] Farrell R. E. Jr. (2009). *RNA Methodologies, A Laboratory Guide for Isolation and Characterization*, 4th Edn, San Diego, CA: Academic Press.

[B18] Hammer Ø. Harper D. Ryan P. (2001). PAST: paleontological statistics software package for education and data analysis. *Palaeontol. Electron.* 4 1–9.

[B19] Howeler M. Ghiorse W. C. Walker L. P. (2003). A quantitative analysis of DNA extraction and purification from compost. *J. Microbiol. Methods* 54 37–45. 10.1016/s0167-7012(03)00006-x 12732420

[B20] James R. H. Green D. R. H. Stock M. J. Alker B. J. Banerjee N. R. Cole C. (2014). Composition of hydrothermal fluids and mineralogy of associated chimney material on the East Scotia Ridge back-arc spreading centre. *Geochim. Cosmochim. Acta* 139 47–71. 10.1016/j.gca.2014.04.024

[B21] Jorgensen S. L. Hannisdal B. Lanzen A. Baumberger T. Flesland K. Fonseca R. (2012). Correlating microbial community profiles with geochemical data in highly stratified sediments from the Arctic Mid-Ocean Ridge. *Proc. Natl. Acad. Sci. U.S.A.* 109 E2846–E2855. 10.1073/pnas.1207574109 23027979 PMC3479504

[B22] Knittel K. Boetius A. (2009). Anaerobic oxidation of methane: progress with an unknown process. *Annu. Rev. Microbiol.* 63 311–334. 10.1146/annurev.micro.61.080706.093130 19575572

[B23] Knittel K. Lösekann T. Boetius A. Kort R. Amann R. (2005). Diversity and distribution of methanotrophic archaea at cold seeps. *Appl. Environ. Microbiol.* 71 467–479. 10.1128/aem.71.1.467-479.2005 15640223 PMC544223

[B24] Kopylova E. Noe L. Touzet H. (2012). SortMeRNA: fast and accurate filtering of ribosomal RNAs in metatranscriptomic data. *Bioinformatics* 28 3211–3217. 10.1093/bioinformatics/bts611 23071270

[B25] Kouduka M. Suko T. Morono Y. Inagaki F. Ito K. Suzuki Y. (2012). A new DNA extraction method by controlled alkaline treatments from consolidated subsurface sediments. *FEMS Microbiol. Lett.* 326 47–54. 10.1111/j.1574-6968.2011.02437.x 22092362

[B26] Lekang K. Thompson E. M. Troedsson C. (2015). A comparison of DNA extraction methods for biodiversity studies of eukaryotes in marine sediments. *Aquat. Microb. Ecol.* 75 15–25. 10.3354/ame01741

[B27] Lever M. A. Torti A. Eickenbusch P. Michaud A. B. Santl-Temkiv T. Jorgensen B. B. (2015). A modular method for the extraction of DNA and RNA, and the separation of DNA pools from diverse environmental sample types. *Front. Microbiol.* 6:476 10.3389/fmicb.2015.00476 PMC4436928 26042110

[B28] Lipp J. S. Morono Y. Inagaki F. Hinrichs K. U. (2008). Significant contribution of Archaea to extant biomass in marine subsurface sediments. *Nature* 454 991–994. 10.1038/nature07174 18641632

[B29] Liu J. Chen M. Chen Z. Yan W. (2010). Clay mineral distribution in surface sediments of the South China Sea and its significance for in sediment sources and transport. *Chin. J. Oceanol. Limnol.* 28 407–415. 10.1007/s00343-010-9057-7

[B30] Lloyd K. G. MacGregor B. J. Teske A. (2010). Quantitative PCR methods for RNA and DNA in marine sediments: maximizing yield while overcoming inhibition. *FEMS Microbiol. Ecol.* 72 143–151. 10.1111/j.1574-6941.2009.00827.x 20059545

[B31] Lloyd K. G. Schreiber L. Petersen D. G. Kjeldsen K. U. Lever M. A. Steen A. D. (2013). Predominant archaea in marine sediments degrade detrital proteins. *Nature* 496 215–218. 10.1038/nature12033 23535597

[B32] Logares R. Sunagawa S. Salazar G. Cornejo-Castillo F. M. Ferrera I. Sarmento H. (2014). Metagenomic 16S rDNA Illumina tags are a powerful alternative to amplicon sequencing to explore diversity and structure of microbial communities. *Environ. Microbiol.* 16 2659–2671. 10.1111/1462-2920.12250 24102695

[B33] Luna G. M. Dell’Anno A. Danovaro R. (2006). DNA extraction procedure: a critical issue for bacterial diversity assessment in marine sediments. *Environ. Microbiol.* 8 308–320. 10.1111/j.1462-2920.2005.00896.x 16423017

[B34] Luo C. Rodriguez R. L. Johnston E. R. Wu L. Cheng L. Xue K. (2014). Soil microbial community responses to a decade of warming as revealed by comparative metagenomics. *Appl. Environ. Microbiol.* 80 1777–1786. 10.1128/AEM.03712-13 24375144 PMC3957593

[B35] Lynch M. D. J. Neufeld J. D. (2015). Ecology and exploration of the rare biosphere. *Nat. Rev. Microbiol.* 13 217–229. 10.1038/nrmicro3400 25730701

[B36] Magoč T. Salzberg S. L. (2011). FLASH: fast length adjustment of short reads to improve genome assemblies. *Bioinformatics* 27 2957–2963. 10.1093/bioinformatics/btr507 21903629 PMC3198573

[B37] Martin-Laurent F. Philippot L. Hallet S. Chaussod R. Germon J. C. Soulas G. (2001). DNA extraction from soils: old bias for new microbial diversity analysis methods. *Appl. Environ. Microbiol.* 67 2354–2359. 10.1128/AEM.67.5.2354-2359.2001 11319122 PMC92877

[B38] Meng J. Xu J. Qin D. He Y. Xiao X. Wang F. (2014). Genetic and functional properties of uncultivated MCG archaea assessed by metagenome and gene expression analyses. *ISME J.* 8 650–659. 10.1038/ismej.2013.174 24108328 PMC3930316

[B39] Miller D. N. Bryant J. E. Madsen E. L. Ghiorse W. C. (1999). Evaluation and optimization of DNA extraction and purification procedures for soil and sediment samples. *Appl. Environ. Microbiol.* 65 4715–4724. 10543776 10.1128/aem.65.11.4715-4724.1999 PMC91634

[B40] Morono Y. Terada T. Hoshino T. Inagaki F. (2014). Hot-alkaline DNA extraction method for deep-subseafloor archaeal communities. *Appl. Environ. Microbiol.* 80 1985–1994. 10.1128/AEM.04150-13 24441163 PMC3957647

[B41] Morono Y. Terada T. Masui N. Inagaki F. (2009). Discriminative detection and enumeration of microbial life in marine subsurface sediments. *ISME J.* 3 503–511. 10.1038/ismej.2009.1 19212428

[B42] Niemi R. M. Heiskanen I. Wallenius K. Lindstrom K. (2001). Extraction and purification of DNA in rhizosphere soil samples for PCR-DGGE analysis of bacterial consortia. *J. Microbiol. Methods* 45 155–165. 10.1016/s0167-7012(01)00253-6 11348673

[B43] Nyyssönen M. Hultman J. Ahonen L. Kukkonen I. Paulin L. Laine P. (2013). Taxonomically and functionally diverse microbial communities in deep crystalline rocks of the Fennoscandian shield. *ISME J.* 8 126–138. 10.1038/ismej.2013.125 23949662 PMC3869007

[B44] Ogram A. Sayler G. S. Barkay T. (1987). The extraction and purification of microbial DNA from sediments. *J. Microbiol. Methods* 7 57–66. 10.1016/0167-7012(87)90025-X

[B45] Ovreas L. Forney L. Daae F. L. (1997). Distribution of bacterioplankton in meromictic Lake Saelenvannet, as determined by denaturing gradient gel electrophoresis of PCR-amplified gene fragments coding for 16S rRNA. *Appl. Environ. Microbiol.* 63 3367–3373. 9292986 10.1128/aem.63.9.3367-3373.1997 PMC168642

[B46] Pinto A. J. Raskin L. (2012). PCR biases distort bacterial and archaeal community structure in pyrosequencing datasets. *PLoS ONE* 7:e43093 10.1371/journal.pone.0043093 PMC3419673 22905208

[B47] Quast C. Pruesse E. Yilmaz P. Gerken J. Schweer T. Yarza P. (2013). The SILVA ribosomal RNA gene database project: improved data processing and web-based tools. *Nucleic Acids Res.* 41 D590–D596. 10.1093/nar/gks1219 23193283 PMC3531112

[B48] Rappe M. S. Giovannoni S. J. (2003). The uncultured microbial majority. *Annu. Rev. Microbiol.* 57 369–394. 10.1146/annurev.micro.57.030502.090759 14527284

[B49] Roose-Amsaleg C. L. Garnier-Sillam E. Harry M. (2001). Extraction and purification of microbial DNA from soil and sediment samples. *Appl. Soil. Ecol.* 18 47–60. 10.1016/S0929-1393(01)00149-4

[B50] Rosa M. Dias R. da Graca Miguel M. Lindman B. (2005). DNA-cationic surfactant interactions are different for double- and single-stranded DNA. *Biomacromolecules* 6 2164–2171. 10.1021/bm050137n 16004459

[B51] Scupham A. J. Jones J. A. Wesley I. V. (2007). Comparison of DNA extraction methods for analysis of turkey cecal microbiota. *J. Appl. Microbiol.* 102 401–409. 10.1111/j.1365-2672.2006.03094.x 17241345

[B52] Sergeant M. J. Constantinidou C. Cogan T. Penn C. W. Pallen M. J. (2012). High-throughput sequencing of 16S rRNA gene amplicons: effects of extraction procedure, primer length and annealing temperature. *PLoS ONE* 7:e38094 10.1371/journal.pone.0038094 PMC3362549 22666455

[B53] Sogin M. L. Morrison H. G. Huber J. A. Mark Welch D. Huse S. M. Neal P. R. (2006). Microbial diversity in the deep sea and the underexplored “rare biosphere”. *Proc. Natl. Acad. Sci. U.S.A.* 103 12115–12120. 10.1073/pnas.0605127103 16880384 PMC1524930

[B54] Song Z. Q. Wang F. P. Zhi X. Y. Chen J. Q. Zhou E. M. Liang F. (2013). Bacterial and archaeal diversities in Yunnan and Tibetan hot springs, China. *Environ. Microbiol.* 15 1160–1175. 10.1111/1462-2920.12025 23126508

[B55] Tebbe C. C. Vahjen W. (1993). Interference of humic acids and DNA extracted directly from soil in detection and transformation of recombinant DNA from bacteria and a yeast. *Appl. Environ. Microbiol.* 59 2657–2665. 7690221 10.1128/aem.59.8.2657-2665.1993 PMC182335

[B56] Teske A. Sorensen K. B. (2007). Uncultured archaea in deep marine subsurface sediments: have we caught them all? *ISME J*. 2 3–18. 10.1038/ismej.2007.90 18180743

[B57] Valentine D. L. (2007). Adaptations to energy stress dictate the ecology and evolution of the Archaea. *Nat. Rev. Microbiol.* 5 316–323. 10.1038/nrmicro1619 17334387

[B58] Vigneron A. Cruaud P. Roussel E. G. Pignet P. Caprais J. C. Callac N. (2014). Phylogenetic and functional diversity of microbial communities associated with subsurface sediments of the Sonora Margin, Guaymas Basin. *PLoS ONE* 9:e104427 10.1371/journal.pone.0104427 PMC4123917 25099369

[B59] Vishnivetskaya T. A. Layton A. C. Lau M. C. Chauhan A. Cheng K. R. Meyers A. J. (2014). Commercial DNA extraction kits impact observed microbial community composition in permafrost samples. *FEMS Microbiol. Ecol.* 87 217–230. 10.1111/1574-6941.12219 24102625

[B60] Vorhies J. S. Gaines R. R. (2009). Microbial dissolution of clay minerals as a source of iron and silica in marine sediments. *Nat. Geosci.* 2 221–225. 10.1038/ngeo441

[B61] Wang F. Zhou H. Meng J. Peng X. Jiang L. Sun P. (2009). GeoChip-based analysis of metabolic diversity of microbial communities at the Juan de Fuca Ridge hydrothermal vent. *Proc. Natl. Acad. Sci. U.S.A.* 106 4840–4845. 10.1073/pnas.0810418106 19273854 PMC2660763

[B62] Wang P. Li T. Hu A. Wei Y. Guo W. Jiao N. (2010). Community structure of archaea from deep-sea sediments of the South China Sea. *Microb. Ecol.* 60 796–806. 10.1007/s00248-010-9746-y 20886337

[B63] Wilson I. G. (1997). Inhibition and facilitation of nucleic acid amplification. *Appl. Environ. Microbiol.* 63 3741–3751. 9327537 10.1128/aem.63.10.3741-3751.1997 PMC168683

[B64] Yeates C. Gillings M. R. Davison A. D. Altavilla N. Veal D. A. (1998). Methods for microbial DNA extraction from soil for PCR amplification. *Biol. Proced. Online* 1 40–47. 10.1251/bpo6 12734590 PMC140122

[B65] Zhang X. Feng X. Wang F. (2016). Diversity and metabolic potentials of subsurface crustal microorganisms from the western flank of the Mid-Atlantic Ridge. *Front. Microbiol.* 7:363 10.3389/fmicb.2016.00363 PMC4797314 27047476

[B66] Zhou J. Bruns M. A. Tiedje J. M. (1996). DNA recovery from soils of diverse composition. *Appl. Environ. Microbiol.* 62 316–322. 8593035 10.1128/aem.62.2.316-322.1996 PMC167800

[B67] Zoetendal E. G. Ben-Amor K. Akkermans A. D. Abee T. de Vos W. M. (2001). DNA isolation protocols affect the detection limit of PCR approaches of bacteria in samples from the human gastrointestinal tract. Syst. *Appl. Microbiol.* 24 405–410. 10.1078/0723-2020-00060 11822677

