# Supplementary material for: A Modified SDS-Based DNA Extraction Method for High Quality Environmental DNA from Seafloor Environments
Source: Front Microbiol. 2016 Jun 23;7:986. doi: 10.3389/fmicb.2016.00986 (PMC4917542; doi:10.3389/fmicb.2016.00986)
Supplement: Supplementary file 1 [file Data_Sheet_1.DOCX]

***Supplementary Material***

**­­­**

**A modified SDS-based DNA extraction method for high quality environmental DNA from seafloor environments**

**Vengadesh perumal Natarajan^1,2#^, Xinxu Zhang^3#^, Yuki Morono^4^, Fumio Inagaki^4^, Fengping Wang^1,2*^**

^1^ State Key Laboratory of Microbial Metabolism, School of Life Sciences and Biotechnology, Shanghai Jiao Tong University, Shanghai, 200240, People’s Republic of China.

^2^ State Key Laboratory of Ocean Engineering, Shanghai Jiao Tong University, Shanghai, 200240, People’s Republic of China.

^3^ Guangdong Provincial Key Laboratory of Marine Biology, Marine Biology Institute, Shantou University, Shantou, 515063, People’s Republic of China.

^4^ Geomicrobiology Group, Kochi Institute for Core Sample Research, Japan Agency for Marine-Earth Science and Technology (JAMSTEC), Monobe B200, Nankoku, Kochi 783-8502, Japan.

^#^ These authors contributed equally to this work.

**^*^ Corresponding author:** Fengping Wang, [fengpingw@sjtu.edu.cn](mailto:fengpingw@sjtu.edu.cn).


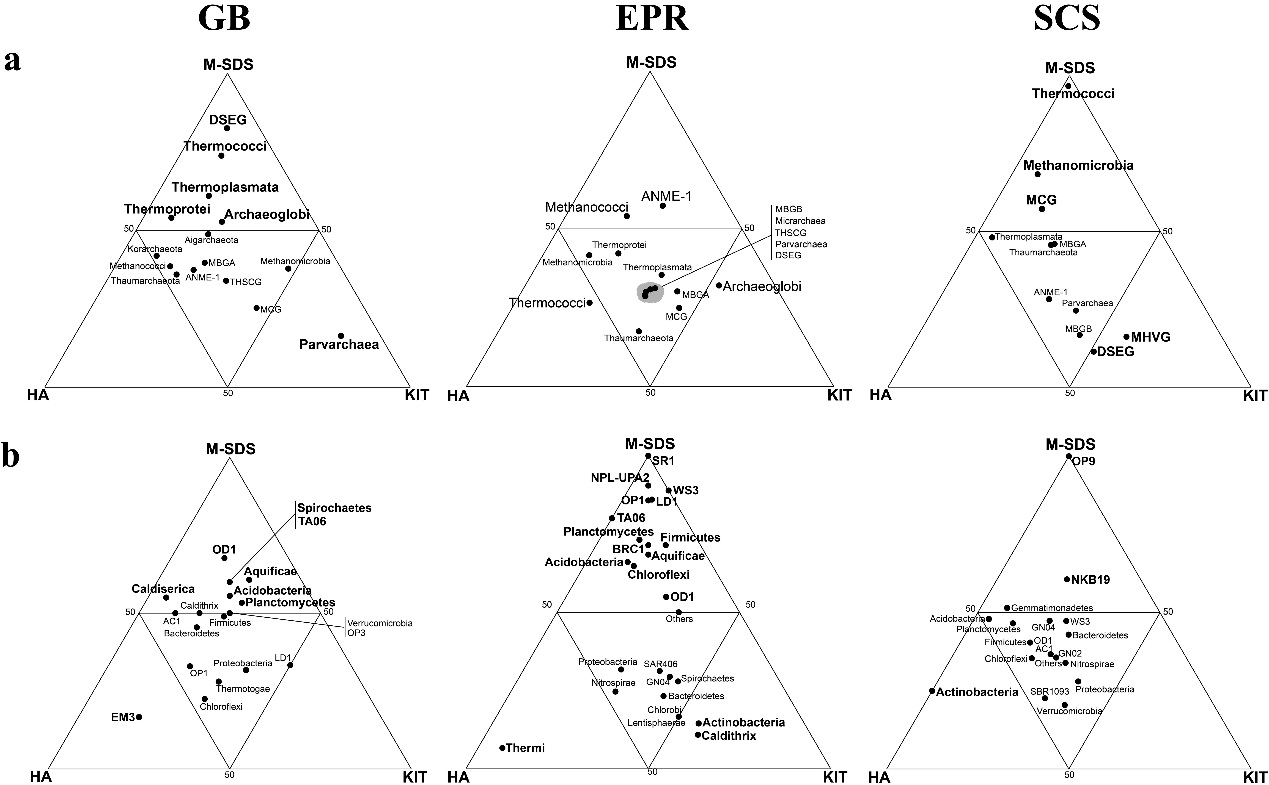


**Figure S1. Ternary plots for (a) archaeal groups (class level) and (b) bacterial groups (phylum level) in three seafloor samples with respect to the DNA extraction methods.** Axes indicate the percentage of the designated groups detected by each method. The groups with more than 50% relative abundance among three methods are in bold font.


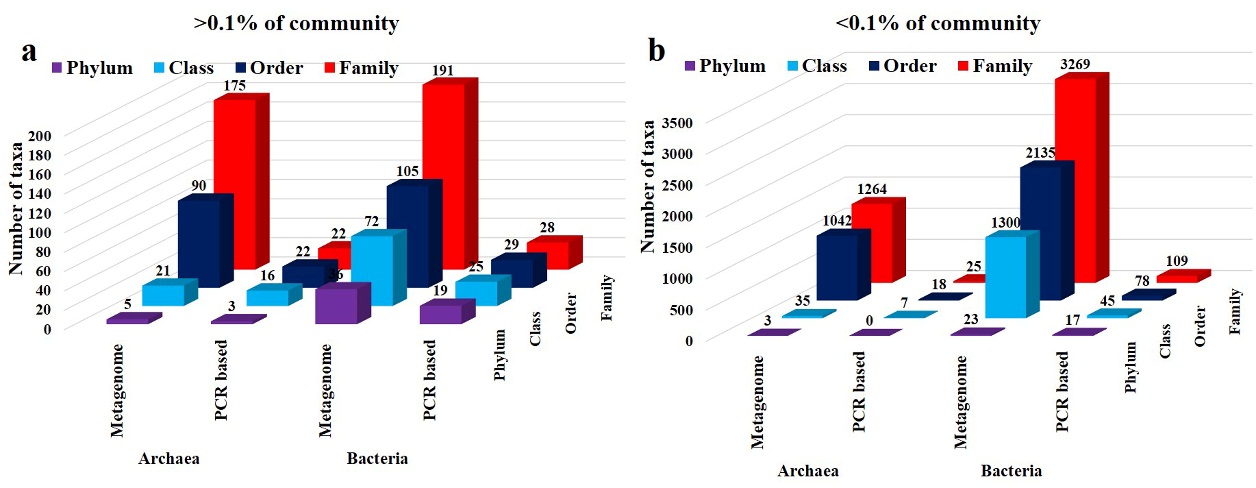


**Figure S2. Comparison of microbial groups from the metagenome data and the PCR-amplified data using the GB DNA sample extracted by the M-SDS method.** **(a)** Archaeal and bacterial taxonomic composition at different taxonomic level, when the designated group is >0.1%. **(b)** Archaeal and bacterial rare biosphere at different taxonomic level, when the designated group is <0.1%.
